# Investigating the relationship of DNA methylation with mutation rate and allele frequency in the human genome

**DOI:** 10.1186/1471-2164-13-S8-S7

**Published:** 2012-12-17

**Authors:** Junfeng Xia, Leng Han, Zhongming Zhao

**Affiliations:** 1 Department of Biomedical Informatics, Vanderbilt University School of Medicine, Nashville, TN 37232, USA; 2 Department of Psychiatry, Vanderbilt University School of Medicine, Nashville, TN 37232, USA; 3 Department of Cancer Biology, Vanderbilt University School of Medicine, Nashville, TN 37232, USA; 4 Center for Quantitative Sciences, Vanderbilt University Medical Center, Nashville, TN 37232, USA

## Abstract

**Background:**

DNA methylation, which mainly occurs at CpG dinucleotides, is a dynamic epigenetic regulation mechanism in most eukaryotic genomes. It is already known that methylated CpG dinucleotides can lead to a high rate of C to T mutation at these sites. However, less is known about whether and how the methylation level causes a different mutation rate, especially at the single-base resolution.

**Results:**

In this study, we used genome-wide single-base resolution methylation data to perform a comprehensive analysis of the mutation rate of methylated cytosines from human embryonic stem cell. Through the analysis of the density of single nucleotide polymorphisms, we first confirmed that the mutation rate in methylated CpG sites is greater than that in unmethylated CpG sites. Then, we showed that among methylated CpG sites, the mutation rate is markedly increased in low-intermediately (20-40% methylation level) to intermediately methylated CpG sites (40-60% methylation level) of the human genome. This mutation pattern was observed regardless of DNA strand direction and the sequence coverage over the site on which the methylation level was calculated. Moreover, this highly non-random mutation pattern was found more apparent in intergenic and intronic regions than in promoter regions and CpG islands. Our investigation suggested this pattern appears primarily in autosomes rather than sex chromosomes. Further analysis based on human-chimpanzee divergence confirmed these observations. Finally, we observed a significant correlation between the methylation level and cytosine allele frequency.

**Conclusions:**

Our results showed a high mutation rate in low-intermediately to intermediately methylated CpG sites at different scales, from the categorized genomic region, whole chromosome, to the whole genome level, thereby providing the first supporting evidence of mutation rate variation at human methylated CpG sites using the genome-wide sing-base resolution methylation data.

## Background

Germline mutation can profoundly affect genetic variation within populations and divergence between species. The identification of factors that modulate mutation rates in germ cells, particularly in humans, has been a long-standing scientific pursuit [1]. Several factors are thought to be related to mutation rate, including DNA replication timing [2], genetic recombination [3], and categorized genomic regions [4]. The mutation rate is also known to vary throughout the genome in a contextual fashion [1], the most well-studied example being the influence of CpG dinucleotides in mammalian genomes. CpGs are generally methylated in mammalian genomes, and since the deamination rates of methylated cytosine is 2.0 to 3.2 fold more than unmethylated cytosine [5], it leads to a 10- to 50-fold higher transition rate of methylated CpG to TpG than other transitional changes [6,7]. This rate difference in turn causes the suppression of CpG sites in the mammalian genomes [8,9]. However, whether and how a variation in methylation level causes the variation in the high mutation rate is still unknown.

Based on the analysis of single nucleotide polymorphisms (SNPs), Fryxell and Moon [10] have concluded that methylation-dependent transition rates are exponentially dependent on local GC content. Our re-examined studies further indicated that these methylation-dependent transition rates in the human genome were dependent on both the local sequence length and the genomic region [7]. In a recent study of CpG mutability in intronic regions of the human genome [11], CpG substitution rates were observed to be significantly correlated with the extent of methylation level. In the same study, they also found that the CpG substitute rate is positively correlated with non-CpG divergence and negatively correlated with GC content [11]. However, their conclusion is based on MeDIP methylation data, which is calculated in a low resolution for a 100-bp (base pair) window size [12]. Moreover, although their results showed that the methylation level affected the mutation rate, their investigation limited in the intronic regions only, and the mutation pattern in other genomic regions remained unclear.

More recently, next-generation sequencing has provided single-base resolution DNA methylome in the human genome [13], thus making more comprehensive analysis possible [14-17]. The availability of whole-genome methylation maps with single-nucleotide resolution provides an opportunity to refine studies pertaining to CpG mutation rate variation. Compared with array data, the advantage of sequencing data can not only be used to estimate methylation broadness but also be used to analyze methylation deepness for each single base [17].

Here, we used genome-wide, single-base-resolution maps of methylation data [13] in human embryonic stem cell line (H1) to perform an extensive investigation of the correlation between the level of cytosine methylation and CpG mutability of the human genome. In addition to the methylation on CpG sites, we also examined the effect of methylation level on non-CpG sites (e.g., CHG/CHH, where H = A, C, or T) sites. We observed that among methylated CpG sites, the mutation rate, as reflected by the human SNP density, is markedly increased in low-intermediately to intermediately methylated CpG sites at different scales, from the categorized genomic region, whole chromosome, to the whole genome level. This observation was also confirmed by human-chimpanzee divergence data. Finally, we found a significant correlation between methylation level and cytosine allele frequency in the human genome.

## Materials and methods

### DNA methylation profile in the human genome

The single-base resolution DNA methylation data were derived from Lister *et al. *[13], including whole genome bisulfite sequencing data for two human cell lines: H1 human embryonic stem cell and IMR90 fetal lung fibroblast. We removed all methylcytosines that could not be mapped to reference CpG sites (NCBI Build 36) in our analysis. For the remaining mapped methylcytosines, we used methylation deepness [17] to evaluate the methylation level in the CpG site, which represents the extent of the methylation level of the cytosines based on the ratio of the number of reads having methylated cytosine over the total number of reads mapped to each reference CpG site. CpG sites can be classified as methylated CpG sites if there is at least one read covering methylated cytosine (methylation level > 0%). Otherwise, they are classified as the unmethylated CpG sites, i.e. methylation level = 0%. Methylated CpG sites are further categorized into five groups of increasing methylation levels: lowly methylated (less than or equal to 20%), low-intermediately methylated (20-40%), intermediately methylated (40-60%), high-intermediately methylated (60-80%), and highly methylated (greater than 80%).

### Genome sequence and annotation

The human reference genome sequence (Build 36) was downloaded from NCBI website [18]. Genomic annotations, including the start and end positions of genes and exons, were defined based on the ENSEMBL database [19]. Promoter was defined as an interval of -1500 to +500 bp around the transcriptional start site (TSS) as previously described[20]. A genic region was defined as the transcribed region from the start to the end of transcription sites. An intergenic region was defined as a region in which no known gene was annotated. Similarly, an intronic region was defined as the region in which no known exon was annotated [20]. According to our previous comparisons [21,22], we used Takai and Jones' algorithm [23] to identify CpG islands (hereafter, we abbreviated as CGIs): length ≥ 500 bp, GC content ≥ 55%, and CpG observed/expected (O/E) ratio ≥ 0.65.

### Estimation of mutation rate

To estimate the mutation rates, we followed the approach in previous studies [2,24] to use the data from both human SNP density and human-chimpanzee divergence. We downloaded the human SNPs data from the NCBI dbSNP database (build 130) website [18] and only considered the validated reference SNPs [4]. A total of 16,969,034 validated reference SNPs were available (with reference SNP ID and reference annotation). Among them, 12,236,967 were bi-allelic and uniquely mapped in the genome, which were used for our further analysis. The SNP density is estimated by computing the total number of C/A, C/G, and C/T SNPs over the total number of cytosine bases in the human genome.

We estimated the divergence of CpGs between the human and chimpanzee genomes based on the human and chimpanzee alignment. We downloaded alignments of the hg18 version of the human genome and PanTro2 version of the chimpanzee genome from UCSC Genome Browser [25]. The divergence is calculated as the proportion of different sites between human and chimpanzee genomes among the total sites that could be aligned between human and chimpanzee genomes.

### Human allele data

For human allele frequency data, we used CEU (Utah residents with Northern and Western European ancestry from the CEPH collection) population data that we downloaded from UCSC Genome Browser [25]. This data set also includes chimpanzee nucleotide information for each allele set when available; thus, we could determine which allele was the ancestral one based on our previous study [4].

## Results and discussion

### Mutation rate in CpG sites with different methylation level

**Figure 1:**
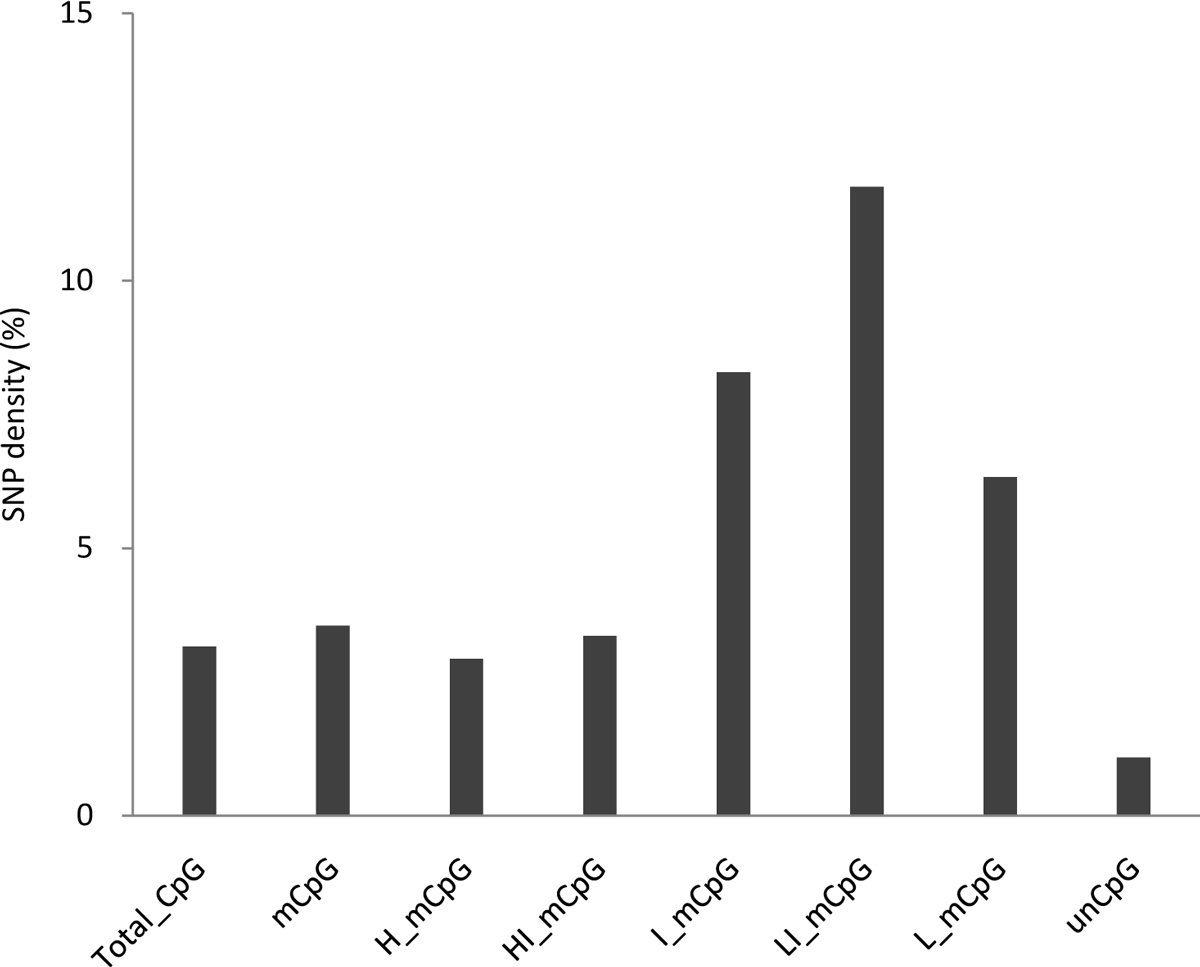
**The mutation rates for all CpG sites, methylated CpG sites and unmethylated CpG sites**. The methylated CpG sites are further categorized into five groups according to their methylation levels. The X-axis includes CpG sites with different methylation levels (Total_CpG: total CpG; mCpG: methylated CpG; H_mCpG: highly methylated CpG (> 80%); HI_mCpG: high-intermediately methylated CpG (60-80%); I_mCpG: intermediately methylated CpG (40-60%); LI_mCpG: low-intermediately methylated CpG (20-40%); L_mCpG: lowly methylated CpG (≤ 20%); unCpG: unmethlated CpG). The Y-axis is the mutation rate that was calculated as the SNP density at CpG sites.

To investigate the mutation rates of CpG sites with different methylation levels, we analyzed the SNP density in the human genome, which represented recent mutations in the human population [2]. We mapped C/A, C/G, and C/T SNPs to CpG sites regardless of their methylation levels. For CpG sites within the same methylation level group, we estimated the mutation rate by computing the number of these SNPs (C/A, C/G, and C/T) over the total number of cytosine bases in the human genome. Since methylated CpGs are highly mutable, one would expect to observe that CpGs with high levels of methylation are more likely to mutate. Indeed, we found that the unmethylated CpGs had a lower mutation rate (1.08%) compared with methylated CpGs (3.55%) (Figure 1). Here, mutation rate was measured by the SNP density [2]. The mutation rate in methylated CpGs was also slightly higher than the total CpGs (3.16%) in the human genome, since more than 80% of CpGs are methylated in the human genome [13]. Among the five CpG methylation level groups, we observed a trend that when CpG methylation level increased, the proportion of CpG sites in that group increased as well (the classification of methylation level was described in the Materials and Methods section). Specifically, the number of CpG sites with lowly, low-intermediately, intermediately, high-intermediately and highly methylated levels was 168,353 (0.7%), 511,909 (2.2%), 1,384,329 (5.9%), 4,557,575 (19.5%), and 16,788,938 (71.7%), respectively. Interestingly, when we compared mutation rate in different groups of methylated CpGs, we found that CpG sites with low-intermediate (20-40%) and intermediate (40-60%) levels of methylation actually had a much higher mutation rate compared with lowly (≤ 20%), high-intermediate (60-80%) and highly methylated CpGs (> 80% methylation). For example, low-intermediately methylated CpG sites had a high mutation rate that could reach up to 11.75%, whereas highly methylated and lowly methylated CpGs only had a mutation rate of 2.93% and 6.32%, respectively.

We compared the mutation rate of methylated CpG sites in H1 and IMR90 cell lines (see additional file 1). We found that the mutation pattern in IMR90 cell line is overall similar to that in H1 cell line, though it is not as evident as in H1 cell line. One possible reason is that the H1 cell line is derived from human embryonic stem cells while the IMR90 cell line is originated from human fetal lung fibroblasts. It has been suggested that stem cells have more opportunity to accumulate mutations compared with mature cells [26]. Therefore, the mutation pattern shows stronger difference in CpG sites with 20-60% methylation level in H1 cell line than IMR90 cell line.

To examine whether the strand bias affected the mutation pattern, we further analyzed the data in the minus strand to compensate for the plus strand pattern observed above. We observed the same pattern when the methylation data of the minus strand was analyzed (see additional file 2). This observation is consistent with the previous report that the DNA methylation levels of two strands in any given genomic region are highly symmetric [13].

The methylation profile we used in this study was generated by single-base resolution bisulfite sequencing technology. According to this technology, the more reads that cover the CpG site, the higher confidence we can obtain regarding the base it detects. Therefore, we checked whether our observation was affected by the read coverage. We found an overall similar pattern regardless of the cut-off values of the coverage we used (see additional file 3). Particularly for CpG sites with low-intermediate and intermediate levels of methylation, we found an even higher mutation rate with higher coverage. For example, we observed that the mutation rate increased to 15.26% when intermediately methylated CpG sites were covered by at least 10 sequencing reads compared with 11.00% when they were covered by at least 5 reads. This finding suggests that our observation that CpG sites with low-intermediate and intermediate levels of methylation actually had a much higher mutation rate is not affected by the sequence coverage of methylated sites. With the increased coverage, our observations could be even more apparent.

### Mutation rate of methylated CpG site in the categorized genomic regions

**Figure 2:**
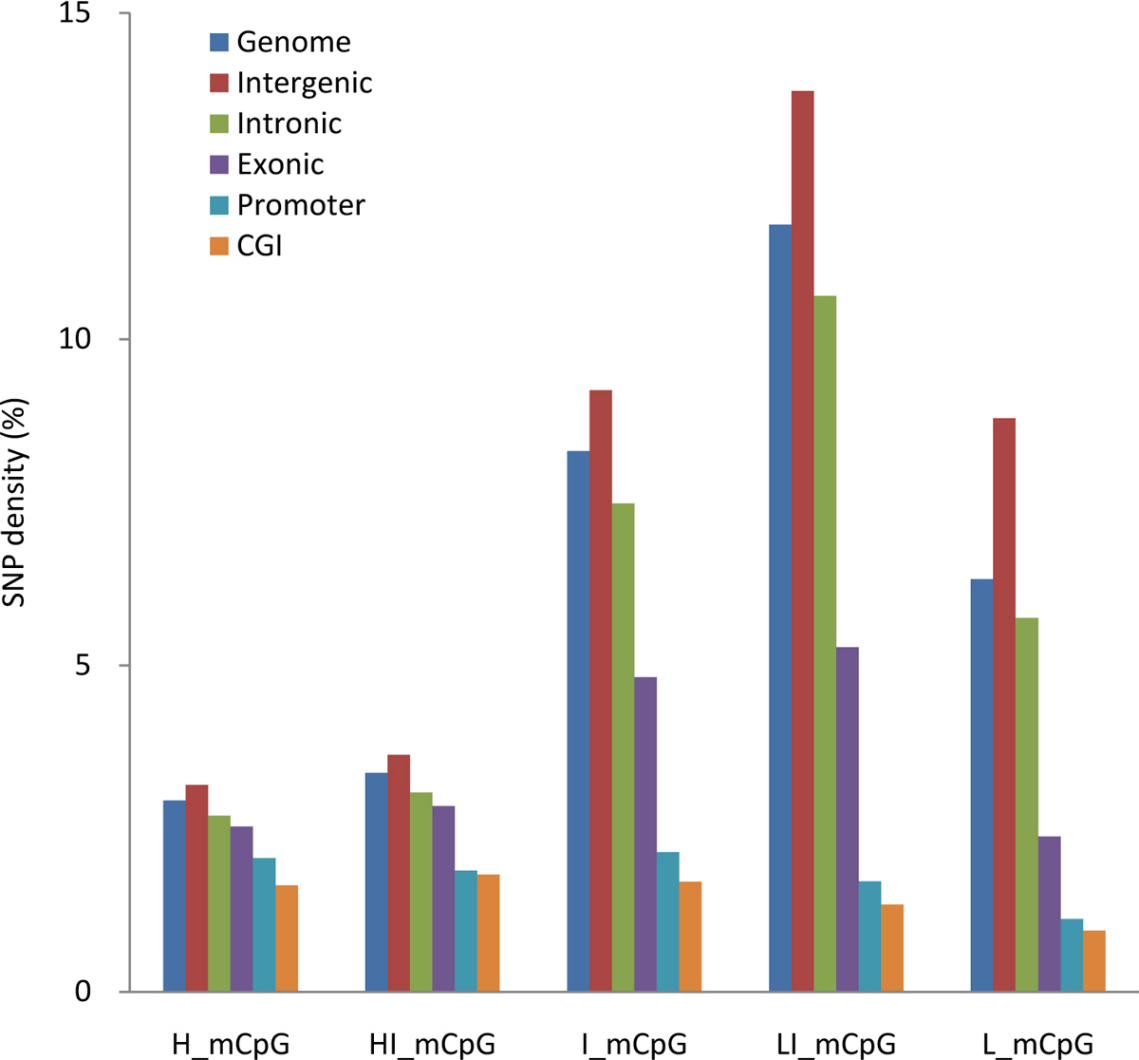
**The mutation rates for methylated CpG sites in the human genome and categorized genomic regions**. The X-axis includes CpG sites with different methylation levels (H_mCpG: highly methylated CpG (> 80%); HI_mCpG: high-intermediately methylated CpG (60-80%); I_mCpG: intermediately methylated CpG (40-60%); LI_mCpG: low-intermediately methylated CpG (20-40%); L_mCpG: lowly methylated CpG (≤ 20%)). The Y-axis is the mutation rate that was calculated as the density of SNPs at CpG sites.

We previously observed the correlation of the rate of germ line mutation with the categorized genomic regions [4]. Here, we further investigated the mutation rates of methylated CpG sites in categorized genomic regions. The results (see Figure 2) are summarized in the following four points. First, we observed a higher distribution of SNP density in intergenic regions compared with other regions of methylated CpG sites at all methylation levels. Here, intergenic regions are defined as regions without any gene annotation. This result was expected, as most mutations in intergenic regions are not subject to selection, and thus, display a higher mutation rate. Second, compared with intergenic regions, the mutation rates in other genomic regions (e.g., intronic regions, exonic regions, promoter regions, and CGIs) were moderately lower than those of the whole genome regardless of their methylation level. Third, for those CpG sites located in the CGIs or promoter regions, the mutation rate was overall very low, but consistent, regardless of the methylation level. This trend is likely due to the lack of methylation and the suppression of methylcytosine deamination in CGIs and promoter regions [4]. Finally, the CpG sites in intergenic regions and intronic regions showed a higher mutation rate with low-intermediate and intermediate levels of methylation.

### Mutation rate of methylated CpG site among chromosomes

**Figure 3:**
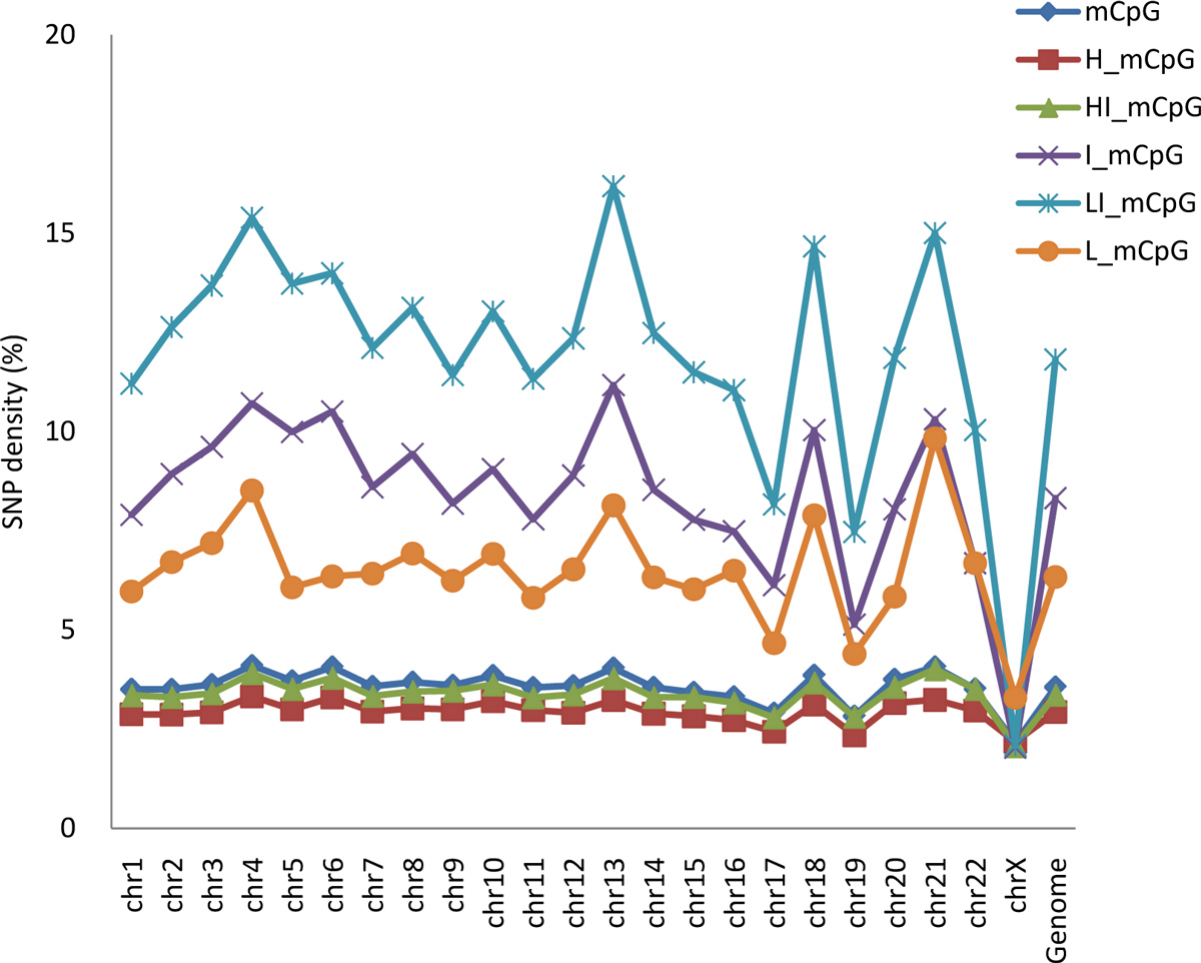
**Comparison of the mutation rates at methylated CpG sites in the human genome and its chromosomes**. Refer to Figure 1 legend for interpretation of abbreviations.

We further examined the mutation rate pattern in human chromosomes (Figure 3). It is already known that the mutation rate varies among chromosomes. Within chromosomes, we observed that CpG sites with low-intermediate and intermediate levels of methylation had a higher mutation rate in all autosomes compared with that of other methylated CpG sites. Furthermore, there were differences in the mutation rate among autosomes. For example, methylated CpG sites in chromosomes 4, 13, 18, and 21 had a higher mutation rate compared with that in other autosomes. We noticed that these four autosomes have overall low gene densities; thus, we further analyzed the relationship between the mutation rate and gene density. We found a negative correlation between the mutation rate and gene density (see additional file 4), and this result further supports the higher mutation rate at the intergenic regions.

A similar mutation pattern could not be observed in the X chromosome due to an overall low mutation rate in this sex chromosome. We only found a slightly higher mutation rate in the CpG sites with a lowly methylated level (0-20%) (Figure 3).

### Mutation rate of non-CpG methylated cytosines

The H1 cell line has abundant non-CpG methylation (mCHG and mCHH, where H = A, C or T) that accounted for nearly 25% of all methylated cytosines. Therefore, we investigated the mutation rate in these methylated non-CpG sites. A summary of the mutation rate from mCHG and mCHH sites was provided in additional file 5. We found that the mutation rates in methylated non-CpG sites were low across the different methylation levels in these sites (0.38-0.42% for mCHG, and 0.39-0.54% for mCHH); thus, we did not observe the pattern as in the methylated CpG sites.

### Sequence divergence at methylated CpG sites

**Figure 4:**
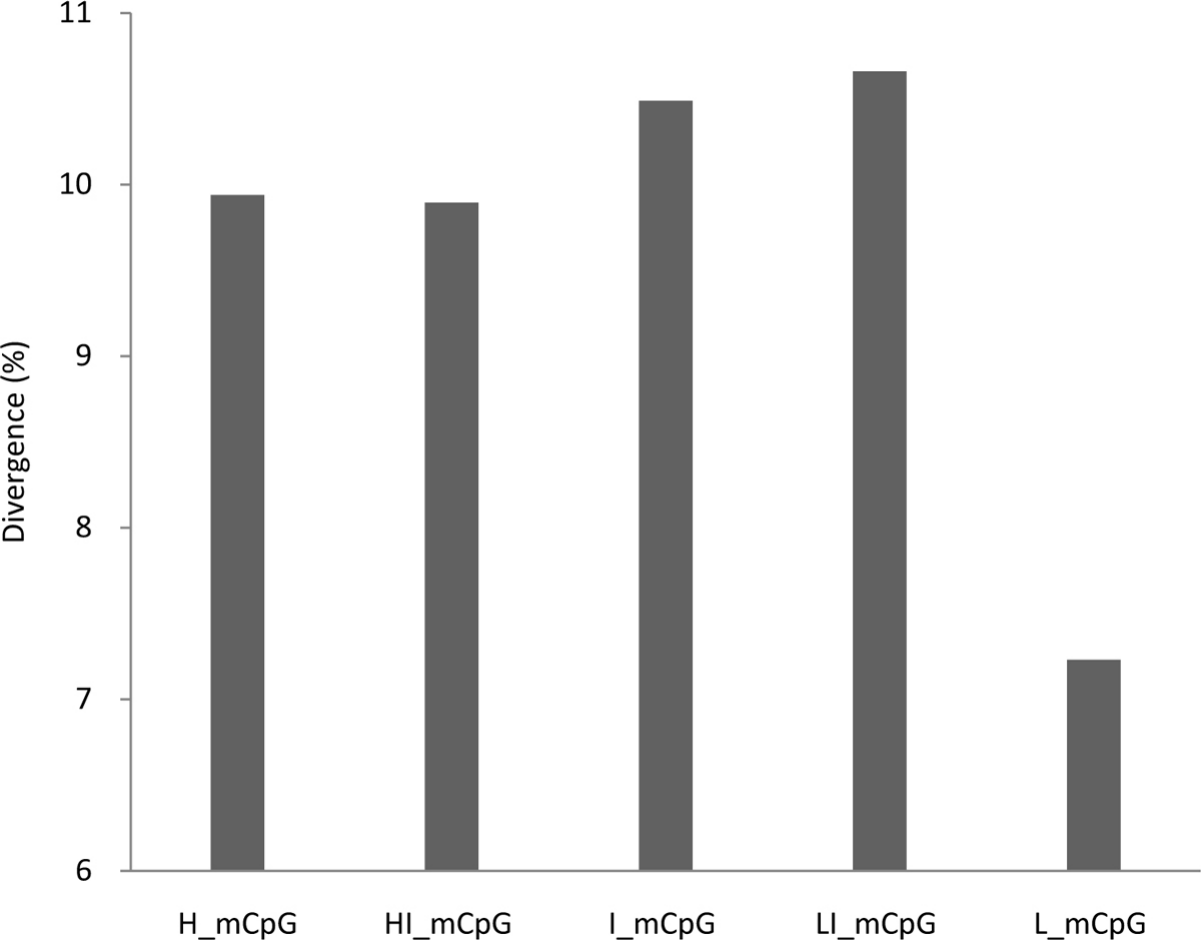
**Human-chimpanzee divergence in CpG sites categorized by different methylation level**. Refer to Figure 1 legend for interpretation of abbreviations.

To further confirm our observations based on SNP density, we used human-chimpanzee sequence divergence to infer the mutation rate (Figure 4). Again, we found the highly methylated CpG sites (9.94%) had larger divergence than lowly methylated CpG sites (7.23%). More interestingly, the divergence of CpGs with methylation levels of 20-40% and 40-60% was 10.66% and 10.49%, respectively. This divergence was higher than the divergence of CpGs with methylation levels of 60-80% and 80-100%, which is consistent with our SNP density data. To exclude the possible effects of selection on functional regions, we further investigated the divergence in intergenic regions. A similar, but even more evident, pattern was observed (see additional file 6).

### Allele frequency at methylated CpG sites

**Figure 5:**
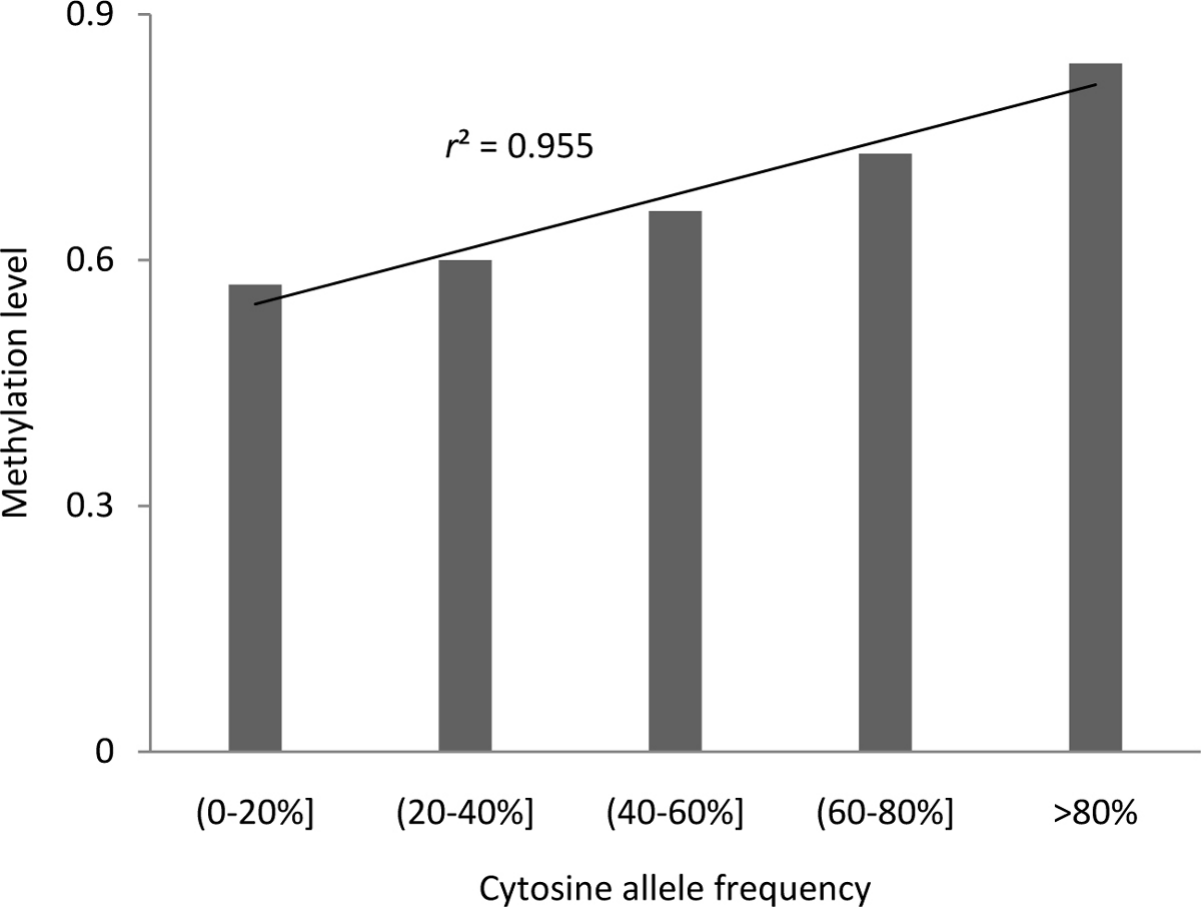
**The correlation between methylation level and cytosine allele frequency**. The X-axis includes five classes of CpG sites defined on the basis of their cytosine allele frequency. The Y-axis is the methylation level that was computed as the number of methylated reads over the total number of reads mapped to each cytosine base. The correlation coefficient (*r*^2^) is shown on the plot.

Using CEU population data from the HapMap Project (release 21), we assigned an allele frequency for each SNP. After mapping C/A, C/G, and C/T SNPs to methylated CpG sites, the ancestor allele of a methylated cytosine site could be inferred by comparing a SNP with its chimpanzee ancestral allele based on our previous method [4]. There were 250,541 methylated CpG sites with inferred ancestor alleles. By calculating the correlation between the methylation level and allele frequency in these methylated CpG sites, we found a significant Pearson correlation between methylation level and cytosine allele frequency (*r = *0.426, *P *< 2.2E-16) as compared to the correlation between methylation level and major allele frequency (*r = *0.198, *P *< 2.2E-16) or the correlation between methylation level and ancestor allele frequency (*r = *0.359, *P *< 2.2E-16). We further binned CpG sites into five classes according to cytosine allele frequency and carried out a regression analysis using methylaton level data. The methylation level associated with the cytosine allele frequency displayed a strong linear dependence with a correlation coefficient *r *= 0.977 (Figure 5).

To further test whether the methyltion level depends on the allele frequency information, we examined the methylation level in CpG sites with different allele frequencies. First, we compared the methylation level when the major allele is cytosine with that when it is not. The methylation level was higher with cytosine as the major allele than with other bases as the major allele (0.798 vs. 0.605, *P *< 2.2E-16). The methylation level was also higher when the ancestral allele is cytosine than other bases (0.792 vs. 0.703, *P *< 2.2E-16). The directionality observed in these values is consistent with the fact that deamination of methylated cytosine results in C → T mutations that likely remain unrepaired and, therefore, become polymorphisms. For those alleles with cytosine as the ancestral allele, we further categorized them as to whether their current major allele is cytosine or other base. We found that for those alleles with cytosine as the ancestor, their methylation level significantly increased when cytosine became the current major allele relative to another base as the current major allele (0.810 vs. 0.610, *P *< 2.2E-16). The increased methylation level of CpG sites with cytosine as both the ancestral allele and the current major allele further suggest that these methylated CpGs are representative of hypermutable sites. Taking together, these results indicated that the subset of methylated CpGs in the derived allele dataset is not a random subset of the genome. The observed overrepresentation of methylated CpGs in all data sets suggests that the methylated CpGs variable is indicative of the previously known hypermutability of methylated cytosines.

## Conclusion

Previous studies have suggested that methylated CpG sites correlate strongly to a high rate of mutation. However, whether and how the higher methylation level causes the higher mutation rate is largely unknown. Our analysis showed that CpG sites with low-intermediate to intermediate levels of methylation (20-60%) actually had a much higher mutation rate as reflected in recent human nucleotide diversity as compared with CpG sites with other methylation levels at different scales, including the categorized genomic regions, whole chromosome, and the whole genome. Consistently, we observed a similar pattern by using human-chimpanzee divergence data.

We further examined variation in the mutation rate among chromosomes. Within autosomal chromosomes, we observed that CpG sites with low-intermediate and intermediate levels of methylation had a higher mutation rate compared with CpG sites with other methylation levels. We found that autosomes with low gene density tend to have an overall higher mutation rate. Because of male-driven evolution [27] in humans, mutation rates are typically highest on the Y chromosome, intermediate on autosomes, and lowest on the X chromosome. Indeed, the mutation rate was higher on the CpG sites in autosomes than that in the X chromosome. However, we could not estimate the mutation rate in the Y chromosome due to the overall low quality data. Further investigation is necessary when high quality data in this sex chromosome is available.

In summary, our study found a significant correlation between the extent of germ line methylation and the mutation rate at human CpG sites. Specifically, we found that there is a high mutation rate in low-intermediately to intermediately methylated CpG sites at different scales, from the categorized genomic region, whole chromosome, to the whole genome level. Mutation rate has been found to be correlated with proximal DNA methylation patterns [11,28], local GC content[10], local sequence lengths and genomic regions[7], local or regional nucleotide composition, crossover rate, distance to telomeres, chromatin compaction [24], as well as the replication timing [2]. Our analysis provides the first supportive evidence regarding the mutation rate variation at human methylated CpG sites using genome-wide single base resolution methylation data. However, further integrative investigation of genetic factors on the mutation rate and methylation level is warranted. Finally, we have shown that there is a significant correlation between methylation level and cytosine allele frequency in the human genome.

## Competing interests

The authors declare that they have no competing interests.

## Authors' contributions

JX carried out the data analysis and drafted the manuscript. LH designed the project, carried out the data analysis, and participated in drafting the manuscript. ZZ conceived of the study, designed the project, and participated in drafting the manuscript. All authors read and approved the final manuscript.

## Supplementary Material

Additional file 1 **The mutation rates at CpG sites inH1 and IMR90 cell lines**. Refer to Figure 1 legend for interpretation of abbreviation. Click here for file

Additional file 2 **The mutation rates for all CpG sites, methylated CpG sites and unmethylated CpG sites in different DNA strands**. Refer to Figure 1 legend for interpretation of abbreviation. Click here for file

Additional file 3 **The mutation rates for methylated CpG sites that are covered by different levels of sequencing reads**. Refer to Figure 1 legend for interpretation of abbreviation. Click here for file

Additional file 4 **The correlation between mutation rate and gene density for methylated CpG sites at the chromosome level**. Each chromosome is plotted as a dot with its gene density per megabase and SNP density on the X and Y axis, respectively. The correlation coefficient (*r*^2^) is shown on the plot. Click here for file

Additional file 5 **The mutation rates for CHG and CHH sites with different methylation levels**. The X-axis includes CHH and CHG sites with different methylation levels (mC: methylated site; H_mC: highly methylated site (> 80%); HI_mC: high-intermediately methylated site (60-80%); I_mC: intermediately methylated site (40-60%); LI_mC: low-intermediately methylated site (20-40%); L_mC: lowly methylated site (≤ 20%)). The Y-axis is the mutation rate that was calculated as the density of SNPs at CHH and CHG sites. Click here for file

Additional file 6 **Human-chimpanzee divergence for methylated CpG sites in the human genome and intergenic regions**. Refer to Figure 1 legend for interpretation of abbreviation. Click here for file
