# Supplementary material for: Investigating the relationship of DNA methylation with mutation rate and allele frequency in the human genome
Source: BMC Genomics. 2012 Dec 17;13(Suppl 8):S7. doi: 10.1186/1471-2164-13-S8-S7 (PMC3535710; doi:10.1186/1471-2164-13-S8-S7)
Supplement: Additional file 5 — The mutation rates for CHG and CHH sites with different methylation levels. The X-axis includes CHH and CHG sites with different methylation levels (mC: methylated site; H_mC: highly methylated site (> 80%); HI_mC: high-intermediately methylated site (60-80%); I_mC: intermediately methylated site (40-60%); LI_mC: low-intermediately methylated site (20-40%); L_mC: lowly methylated site (≤ 20%)). The Y-axis is the mutation rate that was calculated as the density of SNPs at CHH and CHG sites. [file 1471-2164-13-S8-S7-S5.docx]

Figure S5
