# Supplementary material for: Investigating the relationship of DNA methylation with mutation rate and allele frequency in the human genome
Source: BMC Genomics. 2012 Dec 17;13(Suppl 8):S7. doi: 10.1186/1471-2164-13-S8-S7 (PMC3535710; doi:10.1186/1471-2164-13-S8-S7)
Supplement: Additional file 6 — Human-chimpanzee divergence for methylated CpG sites in the human genome and intergenic regions. Refer to Figure 1 legend for interpretation of abbreviation. [file 1471-2164-13-S8-S7-S6.docx]

Figure S6
